# Factors Influencing COVID-19 Vaccine Acceptance in High Income Countries Prior to Vaccine Approval and Rollout: A Narrative Review

**DOI:** 10.3389/ijph.2022.1604221

**Published:** 2022-02-16

**Authors:** Maryke S. Steffens, Bianca Bullivant, Kasia Bolsewicz, Catherine King, Frank Beard

**Affiliations:** ^1^ National Centre for Immunisation Research and Surveillance, Sydney, NSW, Australia; ^2^ The Children’s Hospital at Westmead Clinical School, Faculty of Medicine and Health, The University of Sydney, Sydney, NSW, Australia

**Keywords:** COVID-19, vaccination, vaccine acceptance, vaccine intention, barriers, facilitators

## Abstract

**Objectives:** Acceptance and high uptake of COVID-19 vaccines continues to be critical for controlling the COVID-19 pandemic. This narrative review aimed to summarise findings on factors influencing acceptance of COVID-19 vaccines in the period leading up to the approval and rollout.

**Methods:** We conducted a narrative review of literature published in 2020 on factors influencing acceptance of hypothetical COVID-19 vaccines in adults in high income countries with well-established health systems.

**Results:** Facilitators of acceptance included confidence in vaccine safety and effectiveness, high COVID-19 disease risk perception and trust in health authorities and other vaccine stakeholders, including government. Barriers included safety and effectiveness concerns, perceived scientific uncertainty, low disease risk perception, and low trust in authorities and other stakeholders.

**Conclusion:** Evidence on facilitators and barriers to COVID-19 vaccine acceptance, at a time prior to vaccine rollout, can help health authorities address hesitancy and may inform approaches to support acceptance of novel pandemic vaccines in the future. Future research should include in-depth qualitative research to gather more nuanced evidence.

## Introduction

Uptake of COVID-19 vaccines continues to be critical for controlling the COVID-19 pandemic and returning society to normal functioning [1, 2]. Acceptance of COVID-19 vaccines is essential to achieve high uptake; low acceptance can compromise uptake if not addressed. This has been the experience with other pandemic vaccines, for example, the 2009 H1N1 influenza pandemic vaccine [3, 4].

As with other vaccines, various factors have potential to affect COVID-19 vaccine acceptance and uptake, including perceptions of safety and effectiveness, perceptions of disease risk, social norms, motivational factors, misinformation, and practical factors [5, 6]. While COVID-19 vaccine uptake has been strong in many high income countries, there are benefits to understanding factors that may play a role more broadly in the acceptance of new vaccines developed for novel viruses and pandemics, especially in the period prior to vaccine approval, when awareness may be low, and individuals may be forming attitudes, beliefs, and intentions that influence later vaccination behaviour [5]. Furthermore, early evidence on facilitators and barriers to COVID-19 vaccine acceptance can inform health authorities’ and other vaccine stakeholders’ responses to hesitancy and refusal of vaccines throughout the life of a vaccination program, for example to support acceptance of COVID-19 booster vaccines, the next generation of COVID-19 vaccines, or vaccination of groups eligible to receive COVID-19 vaccines later than others, for example children. Such evidence can also help authorities maintain sensitivity to people’s questions and concerns about COVID-19 vaccines, and respond in ways that build trust in and acceptance of COVID-19 vaccines, or other novel vaccines in the future.

In light of the above, the aim of this narrative review was to explore themes relating to factors influencing acceptance of COVID-19 vaccines in the period leading up to approval and rollout of the vaccines. We focused on acceptance in adults in high income countries with well-established health systems, including the United States, United Kingdom, Australia, New Zealand, Canada, and European countries. The purpose of the review was to reflect on how this summary of evidence can inform health authority responses, in particular communications, to support acceptance of COVID-19 vaccines.

## Methods

An experienced medical librarian conducted a series of database searches designed to identify literature on COVID-19 vaccine knowledge, misinformation, attitudes and practices among adults, including sub populations such as healthcare workers (HCWs). A series of searches were developed in OVID Medline and adapted for OVID PsycINFO. These databases were chosen to provide a complementary mix of both the broad biomedical literature (including some key psychology and social science journals) as indexed by Medline and more specific psychological literature as included in PsycINFO. Database-specific controlled vocabulary terms including “Coronavirus,” “Coronavirus Infections,” “Vaccines,” and “Health Knowledge, Attitudes, Practice” were used in combination with relevant text words, including specific terms for COVID-19 and SARS-COV-2 and terms representing concepts related to attitudes and vaccine misinformation and vaccine uptake. The searches were conducted between 22 November and December 22, 2020. The Medline search strategies used to underpin this narrative review are available in the Supplementary Materials.

Although the primary focus was on research published in peer-reviewed scientific journals, we also reviewed grey literature identified via the reference lists of articles retrieved through the database searches, as well as relevant research published on preprint servers. We included these types of studies in our search due to the urgency of using research findings to better respond to the COVID-19 pandemic.

We included items meeting the following criteria in the review:

• empirical research
• publication date between January 1, 2020 to December 22, 2020
• full-text article available in English
• adult study subjects
• study conducted in high income countries with well-established health systems

We excluded studies in populations in non-comparable countries with different social, cultural and healthcare contexts, as well as studies that focused on aspects of COVID-19 vaccination not related to acceptance.

**FIGURE 1:**
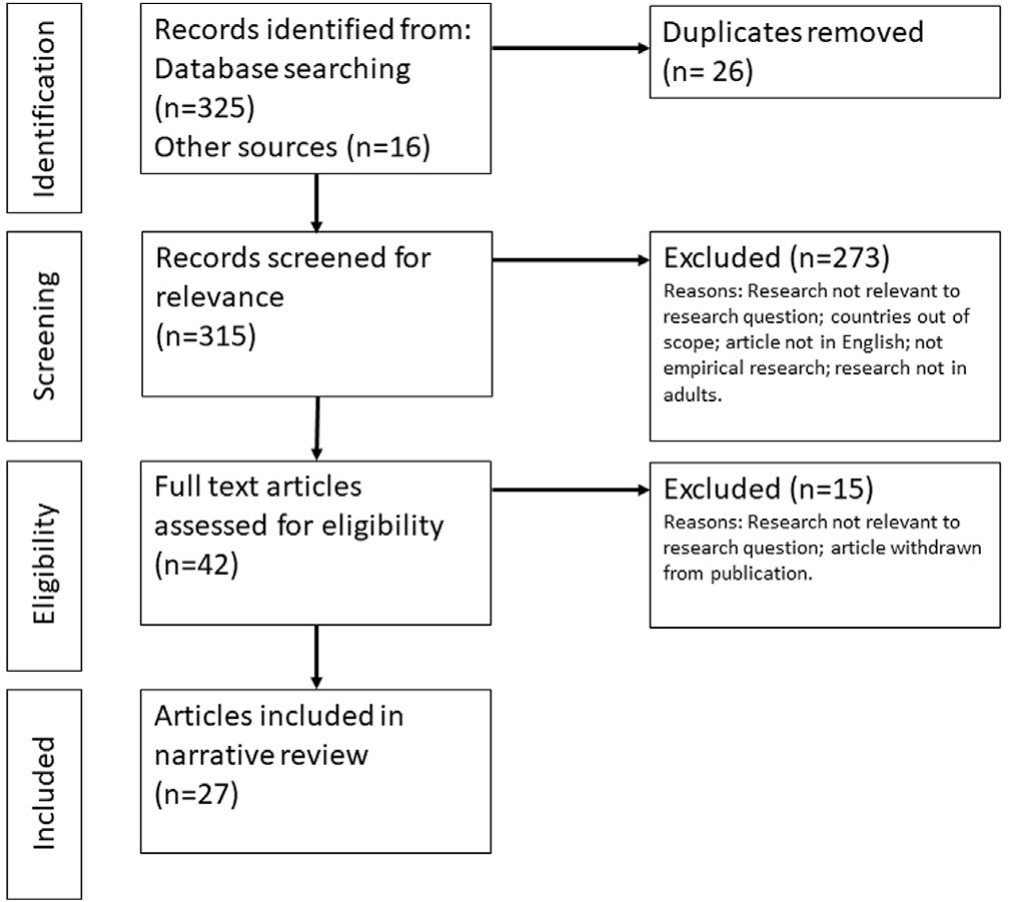
Diagram showing the flow of information through the review (Factors influencing COVID-19 vaccine acceptance in high income countries prior to vaccine approval and rollout: a narrative review; Global, 2020).

Two members of the research team screened 325 titles and abstracts from the initial search and 16 articles and reports identified through other sources, including six preprint articles and 10 grey literature, against the inclusion and exclusion criteria. After screening and removing duplicates, we retrieved 42 papers and reports for a full text review. The final analysis included 27 studies, including 20 articles (including three preprint articles) and seven grey literature reports (see Figure 1).

We intended the review to be primarily descriptive in nature. We extracted data from the articles and summarised the following variables and findings in tabular form using Microsoft Excel: setting (country), study design, population, sample size, month of data collection, as well as sociodemographic characteristics associated with, facilitators of, and barriers to COVID-19 vaccine acceptance. Because vaccine acceptance is described in different ways, we used a range of measures and terminology to indicate vaccine acceptance, including willingness, likelihood or intention to vaccinate; acceptance, confidence and hesitancy, and agreement with positive statements about COVID-19 vaccines.

## Results

### Characteristics of Reviewed Literature

We describe included studies and their characteristics in Table 1.

**TABLE 1:** Characteristics of studies included in the narrative review, stratified by date conducted. (Factors influencing COVID-19 vaccine acceptance in high income countries prior to vaccine approval and rollout: a narrative review; Global, 2020).

Date conducted	Authors	Country	Article type	Method	Population	Sample size	Ref
Feb-20	Papagiannis et al.	Greece	Peer-reviewed article	Personal interview questionnaire	HCWs	461	[13]
Mar-20	Borriello et al.	Australia	Peer-reviewed article	Experiment	Adults	2,136	[15]
Mar-20	Seale et al.	Australia	Peer-reviewed article	Online survey	Adults	1,420	[17]
Mar-20	Detoc et al.	France	Peer-reviewed article	Online survey	Adults, HCWs	3,259 (1,421 HCWs)	[11]
Apr-20	Dodd et al.	Australia	Peer-reviewed article	Online survey	Adults	4,362	[25]
Apr-20	Neumann-Böhme et al.	Denmark, France, Germany, Italy, Portugal, Netherlands, United Kingdom	Peer-reviewed article	Online survey	Adults	7,664	[24]
Apr-20	Williams et al.	United Kingdom	Peer-reviewed article	Online survey	Older adults and people with chronic respiratory disease	527	[9]
Apr-20	Earnshaw et al.	United States	Peer-reviewed article	Online survey	Adults	845	[27]
Apr-20	Fisher et al.	United States	Peer-reviewed article	Online survey	Adults	991	[26]
May-20	Bell et al.	United Kingdom	Peer-reviewed article	Survey, Interviews	Parents	1,252 surveyed 19 interviewed	[7]
May-20	Roozenbeek et al.	United Kingdom, Ireland, United States, Spain, Mexico	Peer-reviewed article	Online survey	Adults	5,000	[18]
May-20	Malik et al.	United States	Preprint article	Online survey	Adults	672	[22]
May-20	NORC	United States	Grey literature	Online survey	Adults	1,056	[21]
May-20	Reiter et al.	United States	Peer-reviewed article	Online survey	Adults	2,006	[20]
May-20	Taylor et al.	United States and Canada	Peer-reviewed article	Online survey	Adults	3,674	[19]
Jun-20	Rhodes et al.	Australia	Peer-reviewed article	Online survey	Parents	2,018	[8]
Jul-20	Lazarus et al.	19 countries	Peer-reviewed article	Online survey	Adults	13,426	[23]
Jul-20	Kreps et al.	United States	Peer-reviewed article	Online survey	Adults	1,971	[29]
Aug-20	Edwards et al.	Australia	Preprint article	Online survey	Adults	3,061	[31]
Aug-20	Dror et al.	Israel	Peer-reviewed article	Online survey	Adults, HCWs	1,941 (829 HCWs)	[12]
Aug-20	IPSOS August 2020	Multiple	Grey literature	Online survey	Adults	1,000	[30]
Sep-20	Pew Research Center	United States	Grey literature	Online survey	Adults	10,093	[28]
Oct-20	IPSOS October 2020	Multiple	Grey literature	Online survey	Adults	18,000	[16]
Oct-20	Gadoth et al.	United States	Preprint article	Online survey	HCWs	609	[14]
Nov-20	Pew Research Center	United States	Grey literature	Online survey	Adults	12,648	[33]
Dec-20	IPSOS December 2020	Multiple	Grey literature	Online survey	Adults	13,500	[32]
Dec-20	KFF	United States	Grey literature	Phone interviews	Adults, HCWs	1,676	[10]

#### Study Participants

The majority (*n* = 19) of studies focused on general adult participants. In terms of subgroups, two studies focused specifically on parents [7, 8] and one study focused on a combination of older adults and people with chronic respiratory conditions [9]. Three studies focused on a combination of adults and healthcare workers [10–12]; and two studies focused exclusively on healthcare workers [13, 14].

#### Study Methods

The majority (*n* = 23) of articles collected data using online cross-sectional surveys. Other study methods included a combination of survey and qualitative in-depth interviews [7]; quantitative phone interviews [10]; a quantitative personal interview questionnaire [13]; and an experimental study design [15].

#### Sample Sizes

Sample sizes varied considerably. Online surveys ranged from 527 participants [9] to global surveys of 18,000 participants [16].

#### Timing of Data Collection

The timing of data collection ranged from February 2020 to December 2020. Most articles (*n* = 13) collected data in the second quarter of 2020 (April–June).

• four were collected in the first quarter of 2020 (January–March) [11, 13, 15, 17];
• 13 were collected in the second quarter of 2020 (April–June) [7–9, 18–27];
• five were collected in the third quarter of 2020 (July–September) [12, 28–31];
• five were collected in the fourth quarter of 2020 (October-December) [10, 14, 16, 32, 33].

### Socio-Demographic Characteristics Associated With COVID-19 Vaccine Acceptance

Various studies found the following socio-demographic characteristics to be significantly associated with lower acceptance of COVID-19 vaccines:

• age <55 years (Australia, United States) [8, 17, 26];
• female gender (United States, Greece, France) [11, 13, 22, 26];
• having a lower household income or socioeconomic status (Australia, United Kingdom) [7, 8, 31];
• having a lower health literacy or education level (Australia, United States) [22, 25, 26, 31];
• living in a rural area (United States) [26].

Various studies found the following socio-demographic characteristics to be significantly associated with higher acceptance of COVID-19 vaccines:

• age 55 or older (multiple countries) [17, 18, 23, 31];
• male gender (Australia, United States, various European countries) [8, 11, 13, 24, 27, 31];
• having a chronic health condition (Australia) [17];
• holding private health insurance (Australia) [17];
• having a liberal political leaning (United States) [20, 28, 33];
• having had a previous influenza vaccination or intending to get a future influenza vaccination, among both healthcare workers (Israel) [12] and adults more broadly (United States) [33];
• among healthcare workers, being a doctor/physician (as opposed to a nurse or paramedic) (Israel, Greece, United States) [12–14].

### Barriers to COVID-19 Vaccine Acceptance

Included articles suggested seven factors acting as potential barriers to COVID-19 vaccine acceptance. We describe these below.

#### Safety Concerns

Vaccine safety concerns were commonly reported as a barrier to COVID-19 vaccine acceptance, including among adults (United States, United Kingdom, Israel, multi-country studies) [9, 10, 12, 16, 19–21, 28, 30, 32], parents (Australia, United Kingdom) [7, 8] and healthcare workers (Israel, United States) [10, 12]. Safety concerns played out in various forms, including concerns about the speed of development and fears that it was rushed and quality could be compromised or safety measures overlooked [7, 9, 12, 28]; the newness of the vaccines [7, 9] and concerns about potential unknown severe or long term side effects [19]; concerns about side-effects in general [9, 10, 12, 16, 20, 21, 30, 32]; and concern about contracting COVID-19 disease from the vaccine [12, 21].

For example, an online survey of 10,093 US adults found that more than three-quarters of respondents were concerned that approval processes were moving too fast (78%) and that COVID-19 vaccines would be approved before their safety was fully understood (77%) [28]. Several studies found a statistical correlation between concerns about future COVID-19 vaccine-related harms and intention not to vaccinate [19, 20]. For example, an online survey of 3,674 American and Canadian adults, conducted in May 2020, found that anxiety around unforeseen future effects of the vaccine was positively correlated with rejection of the vaccine (*p* < 0.001) [19].

#### Doubts About COVID-19 Vaccine Effectiveness

Doubts about how well the vaccine would work in trials (efficacy) and in practice (effectiveness), and how long immunity from the vaccine would last, were also reported as a potential barrier to acceptance of COVID-19 vaccines among adults (United States, Canada) [19–21], parents (Australia, United Kingdom) [7, 8], and healthcare workers (Israel) [12]. For example, a survey of 1,056 North American adults, conducted in May 2020, found that of those people who said they wouldn’t vaccinate, 30 per cent indicated their belief that the vaccines will not work very well as a reason for not vaccinating [21]. In a survey of 2,018 Australian parents conducted in June 2020, 83% of those who were identified as hesitant toward or refusing of COVID-19 vaccines were concerned about vaccine efficacy [8]. Similarly, in a survey of 1,252 United Kingdom parents conducted in April-May 2020, concerns about efficacy were identified as a major reason for not accepting COVID-19 vaccines [7].

#### Perceived Scientific Uncertainty or Wanting Additional Knowledge

In a study of 609 healthcare workers conducted in September-October 2020 in California, United States, respondents cited the perceived evolving and uncertain science around COVID-19 vaccines as a reason for wanting to delay receiving a COVID-19 vaccine [14]. The survey found that of those respondents who intended to delay vaccination (67%), 76% cited concerns about the evolving SARS-CoV-2 science, despite expressing confidence in vaccine safety, effectiveness, and importance for self-protection and community health. Nurses were more than four times more likely to report intent to delay coronavirus vaccine uptake than doctors [14]. A survey of 991 US adults, conducted in April 2020, cited a desire for more information about COVID-19 vaccines as a barrier to COVID-19 vaccine acceptance [26]. This study found that wanting more information was a reason for feeling unsure or not intending on getting vaccinated [26].

#### Low Perceived Risk of Developing Severe COVID-19 Disease

Low perceived risk of developing severe COVID-19 disease was identified as a barrier to acceptance of COVID-19 vaccines among Australian parents, American and Israeli adults, and Israeli healthcare workers [8, 12, 21]. Reasons for hesitancy or intention not to vaccinate in these studies included the view that symptoms of COVID-19 are mild and a vaccine was unnecessary, and a reported lack of concern about becoming seriously ill from COVID-19.

For example, in a survey of 2,108 Australian parents, conducted in June 2020, 27% of participants who were unsure or unwilling to accept a COVID-19 vaccine did not believe a COVID-19 vaccine was necessary [8]. In a survey of 1,056 North American adults, conducted in May 2020, 31% of those people who said they would not vaccinate indicated they were not concerned about getting seriously ill from COVID-19 [21].

#### Doubts About the Seriousness of the Pandemic

Perceived exaggeration of the threat posed by the COVID-19 pandemic by health authorities or the media was identified by several studies as a barrier to acceptance of COVID-19 vaccines (Australia, United States, United Kingdom) [9, 21, 25]. For example, in a survey of 4,362 Australian adults, conducted in April 2020, 44% of those people who said they wouldn’t vaccinate believed the threat of COVID-19 had been exaggerated, compared to 12% of those who said they would get the vaccine and 20% of those who were indifferent [25]. In a survey of 527 United Kingdom older adults and people with chronic respiratory disease, conducted in April 2020, intention to vaccinate was negatively associated with perceiving the media to have overstated the threat of the pandemic [9].

#### Subscribing to COVID-19 Misinformation or Conspiracies

Subscribing to misinformation or conspiracy theories was identified as a barrier to COVID-19 vaccine acceptance among adults in several countries [18, 27]. In an online survey of 845 US adults, conducted in April 2020, those who reported subscribing to conspiracy theories were almost four times less likely to indicate they would receive a COVID-19 vaccine [27]. Examples of conspiracies included the pandemic being a myth to mandate vaccination, government and pharmaceutical companies encouraging the spread of COVID-19 for financial gain, and 5G as the cause of COVID-19 [27]. Subscribing to misinformation was also found to be a barrier to positive intention to vaccinate among a minority of 5,000 individuals surveyed in May 2020 across multiple countries (United Kingdom, Ireland, United States, Spain, Mexico) [18].

#### Lack of Trust in Authorities and Vaccine Stakeholders

A lack of trust in health authorities and suspicion about the motives of other stakeholders involved in vaccine development, such as pharmaceutical companies, was identified as a barrier to accepting COVID-19 vaccines in several studies (United States, Canada) [10, 19, 26]. For example, a survey of 991 US adults, conducted in April 2020, found that participants’ reasons for being unsure or not intending to be vaccinated included a lack of trust in entities involved in vaccine development, testing, or dissemination [26]. A survey of 3,674 US and Canadian adults found that rejection of COVID-19 vaccines was correlated with concerns about commercial profiteering from pharmaceutical companies (*p* < 0.001) [19].

### Facilitators of COVID-19 Vaccine Acceptance

Included articles suggested five factors acting as facilitators of COVID-19 vaccine acceptance. We describe these below.

#### High Perceived Risk of Developing COVID-19 Disease

High perceived risk of developing severe disease was identified as facilitating COVID-19 vaccine acceptance among adults (United Kingdom, United States, Australia) [9, 17, 20, 33] and healthcare workers (France, Israel) [11, 12]. Reasons for high perceived risk included feeling susceptible due to older age, having a chronic health condition, or working in a high-risk occupation [9, 17]. Healthcare workers interacting with COVID-19 patients were more likely to accept COVID-19 vaccination than those who were not (Israel) [12].

For example, an online survey of 527 older United Kingdom adults and people with chronic respiratory disease found that fears about the potential severity of COVID-19 disease on personal health and fears of dying from COVID-19 facilitated intention to receive a COVID-19 vaccine [9]. An online survey of 1,420 Australian adults found that those with a chronic disease were more likely to agree that getting vaccinated would be a good way to protect against COVID-19 infection and disease [17]. In terms of actual risk, a global survey of 13,426 adults in 19 countries found that participants in countries with medium and high incidence and mortality from COVID-19 were more likely to accept COVID-19 vaccines [23].

#### Confidence in Vaccine Effectiveness and Safety

Confidence in COVID-19 vaccine safety and the vaccines’ ability to offer protection, including longer duration of protection and low instances of side effects, were identified as facilitators of COVID-19 vaccine acceptance in adults (Australia, United States, United Kingdom) [7, 9, 15, 19, 20, 29]. For example, in an online survey of 1,971 US adults, conducted in July 2020, modifying characteristics of a hypothetical vaccine, such as increasing efficacy (from 50% to 70% and 50%–90%), increasing protection duration from 1 to 5 years, and decreasing the incidence of adverse effects, was associated with a higher probability of accepting a vaccine [29].

#### Trust in Science and Vaccine Development Processes

Trust in vaccine development processes and scientists was found to facilitate COVID-19 vaccine acceptance among adults (United Kingdom, Ireland, United States, Spain, Mexico) [18, 33]. Adults in a multi-country study were also more likely to recommend the vaccine to others if they trusted scientists [18]. For example, a cross-cultural survey of adults from Ireland, United States, Spain, Mexico, and United Kingdom, conducted in May 2020, found that having a higher trust in scientists was associated with an increased likelihood of getting vaccinated and of recommending others to get vaccinated [18]. An online survey of 12,658 US adults, conducted in November 2020, found that confidence in the research and development process to produce a safe and effective vaccine was associated with increased intention to vaccinate [33].

#### Trust in Government and Health Systems

Trust and confidence in government or health systems was found to be associated with positive COVID-19 vaccine intentions [23, 31]. For example, in a global survey of 13,426 individuals from 19 countries conducted in June 2020, respondents who indicated trust in their government were more likely to accept a COVID-19 vaccine [23]. An online survey of 3,061 Australian adults found that those with confidence in government were less likely to have high levels of hesitancy and more likely to intend to get vaccinated [31].

#### Future Healthcare Provider Recommendation

A US-based study of 2,006 adults found participants were more likely to accept COVID-19 vaccines if they thought their healthcare provider would recommend them [20]. A survey of 1,676 US adults found personal healthcare providers were the most trusted source for information on COVID-19 vaccines [10].

## Discussion

This narrative review of 27 articles and reports explores themes relating to factors influencing acceptance of COVID-19 vaccines in the period leading up to the approval and rollout of the vaccines. Many studies identified concerns about safety and effectiveness as factors influencing COVID-19 vaccine acceptance. These findings are in line with previous findings on other vaccines [5, 34–36]. Concerns about safety and effectiveness, as well as perceived scientific uncertainty and a desire for more information, are perhaps unsurprising given the newness of COVID-19 vaccines and the speed with which they were developed, as well as the use of novel and unfamiliar technology such as mRNA. Other vaccines, such as HPV vaccines, have also inspired similar caution and hesitancy when first introduced [37].

Several studies identified low disease risk perception as a barrier to COVID-19 vaccine acceptance. We hypothesise that this barrier may be relevant in countries where COVID-19 has been politicised, for example the US, or in countries like Australia where the pandemic was well contained for an extended period of time. In contrast, several studies found that high perceived risk of developing COVID-19 disease facilitated COVID-19 vaccine acceptance. Similarly, perception of disease risk has been shown to play a role in motivating people to vaccinate against influenza [38].

Several studies reported trust—in health authorities, in governments, in scientists and the vaccine development process—as a facilitator of COVID-19 vaccine acceptance. Given that uncertainty, exacerbated by rapidly changing public health recommendations, may have characterised many people’s experience of the COVID-19 pandemic more broadly, engendering public trust in public health experts who address uncertainty about vaccines may be particularly important. Some studies found that healthcare provider recommendations were a facilitator of COVID-19 vaccine acceptance; trust may in part explain this finding. In studies of acceptance of routine vaccinations, trust in healthcare providers has been found to support vaccine confidence and acceptance, while primary healthcare providers are consistently found to be the most trusted source of information on routine vaccinations including influenza [39].

### Recommendations

Findings indicate that, at the time of this review, COVID-19 vaccines had not elicited any new types of concerns around vaccine acceptance, with barriers and facilitators presenting in a similar way as for other already established vaccines [40, 41]. This is an important observation because there are several known effective strategies that can be operationalized in response to COVID-19 vaccine concerns, including communication strategies. We note that strategies beyond communication could also be operationalized to improve uptake of COVID-19 vaccines beyond vaccine acceptance, if there are known access barriers to vaccination [41].

The purpose of this review was to explore themes describing factors influencing COVID-19 vaccine acceptance, and to reflect on how this evidence can inform health authority responses, especially communications. This is in keeping with risk communication literature, which recommends using formative research to inform communication efforts [42, 43]. Communications may increase people’s motivation to get vaccinated by addressing what people think and feel, and may increase awareness during the rollout of new vaccines such as COVID-19 [5]. Communications may also mitigate potential threats to vaccine confidence and support acceptance of COVID-19 vaccines throughout the life of a vaccination program.

One factor identified in this narrative review—that safety and effectiveness of new vaccines as well as perceived scientific uncertainty may concern some individuals—suggests that health authorities and other vaccine stakeholders could benefit from adopting approaches aligned with risk communication principles. First, they could be open and forthcoming with information about COVID-19 or other new vaccines and vaccine rollouts. Being transparent with information and providing it in a timely manner can build trust with communities [42, 43]. Second, health authorities and other vaccine stakeholders, such as government and vaccine regulatory authorities, could engage with specific questions and concerns to help reassure communities about vaccine safety and effectiveness. The accelerated trials of COVID-19 vaccines, coupled with a perception that they were rushed, may have heightened safety and effectiveness concerns about COVID-19 vaccines [44]. Research with other vaccines suggests that beliefs about low safety and effectiveness can result in low confidence and acceptance [40, 45]. In terms of approaches, concerns about safety and unanticipated long-term side effects could be addressed by sharing details about the processes in place for ongoing safety monitoring, and emphasizing numbers of people already vaccinated globally and associated safety data. This information could be delivered by credible sources, such as well-known scientists or medical professionals, who are trusted sources of vaccine information [36], including about COVID-19 vaccines specifically [46]. Third, any information should be complemented with opportunities for two-way communication, where individuals can easily ask questions about COVID-19 vaccination, for example via online or public forums, telephone hotlines, or social media. Fourth, any information should be easy to digest, for example using visuals (e.g., infographics), tables or charts to clarify and enhance text. Individuals may not have the time, capacity or desire to digest lengthy research findings on COVID-19 vaccines. Tools that assess readability (e.g., Flesch-Kincaid, FOG or SMOG tests) can simplify and bring clarity to text. Any messaging should be pre-tested with target audiences, as individuals can respond unpredictably to messages about vaccination [47, 48].

Another finding of this review—that low perceived risk of developing severe COVID-19 disease and doubts about the seriousness of the pandemic can be a barrier to vaccine acceptance—suggests that individuals should be supported to understand their personal risk of COVID-19 disease, as well as the risks to the broader community. In general, individuals who perceive themselves at low risk of disease may be less willing to vaccinate [49]. Informing individuals about health consequences is an evidence-based behaviour change technique (BCT) used frequently for persuasion/education in BCT taxonomy [50, 51]. Strategies to address personal risk could include providing information on risks of contracting COVID-19 and suffering severe disease, stratified by age, occupation, and comorbidity, and providing emerging evidence of “long COVID-19” (where symptoms do not resolve for several weeks or months following infection). This information could be framed in terms of anticipated regret; such messages emphasizing anticipated regret of infection have been associated with positive intentions for other vaccines [52–54]. However, individuals may respond unpredictably to appeals to negative emotions and may decrease their intention to vaccinate [5]. Such strategies should therefore be used with caution, and always pre-tested with target groups (including both confident and hesitant individuals). Strategies to address population level risk could include explaining (visually if possible) the concept of exponential growth, i.e. the ability for low numbers of COVID-19 cases to increase quickly to unmanageable levels. This strategy has been shown to encourage support for COVID-19 public health measures [55], and may extend to support for vaccination.

Exposure to misinformation and conspiracies has potential to affect people’s perception of the safety of COVID-19 vaccines [56, 57], which may in turn affect uptake. Debunking misinformation may have a role in supporting vaccine acceptance. Health authorities could identify misinformation that is getting considerable attention, and debunk it by emphasizing factual information, exposing flawed arguments, and providing alternative explanations [58].

### Limitations

The articles and reports included in this review were from high income countries with well-established health systems only. Caution should to be taken when interpreting findings and extrapolating to other contexts, particularly low-income countries. Findings from individual studies offer a snapshot at a particular time and in a particular context. Given rapidly evolving knowledge and circumstances surrounding COVID-19 vaccines, factors influencing COVID-19 vaccine acceptance may fluctuate and change over time.

Our analysis was intended to be primarily descriptive in nature. We did not perform quality assessment of included studies. Studies that were more representative or of higher quality were not given more weight in the analysis. Given the scope of the included studies, we were not able to tease out barriers and facilitators specific to particular vaccines and vaccine technologies, such as mRNA versus viral vector vaccines.

Most studies included in this review report from cross sectional, quantitative surveys. While rapid data collection has been necessary during the COVID-19 pandemic to readily inform the responses of health authorities and other vaccine stakeholders such as regulatory authorities, qualitative interview- or focus group-based research may offer a deeper understanding of COVID-19 vaccine acceptance.

Socio-demographic characteristics associated with vaccine acceptance need to be interpreted with caution. Using individual characteristics, for example being female or a nurse, to predict acceptance can be misleading, as such analyses do not account for the complex factors that influence people’s perspectives and intentions. This approach may also lead to profiling individuals and groups as more or less accepting of COVID-19 vaccines, which may lead to stigma and discrimination. A more nuanced approach is needed, underpinned by an understanding of the broader social, economic, and cultural determinants of COVID-19 vaccine acceptance.

There are inherent limitations with the use of preprint articles and grey literature as these have not been peer-reviewed. This should be borne in mind when interpreting findings. Preprint articles and grey literature used in this review are clearly differentiated from peer-reviewed articles in Table 1.

### Future Research

Future research should use qualitative methods to further explore and understand people’s perspectives on COVID-19 vaccines, prioritising groups more susceptible to infection and/or severe disease, such as health and aged care workers, older adults, and adults with comorbidities, and groups eligible to receive COVID-19 vaccines at later stages of vaccination programs, such as children. Given the dynamic nature of the pandemic, knowledge on the safety and effectiveness of COVID-19 vaccines, and changing policies and public health recommendations, both qualitative and quantitative data on factors influencing COVID-19 vaccine acceptance should be iteratively collected over time. This should include in countries not covered by this review. Analysis of how factors influencing acceptance over time would also be beneficial to inform future efforts to support acceptance of novel pandemic vaccines.

### Conclusion

We found that in high income countries with well-established health systems, factors influencing people’s acceptance of COVID-19 vaccines in the period prior to vaccine approval and rollout include concerns about vaccine safety and effectiveness, trust in health authorities and other vaccine stakeholders, and perceived scientific uncertainty.

We propose potential communication strategies for consideration by health authorities. These include being open and forthcoming with information about COVID-19 vaccines; engaging with specific questions and concerns; and ensuring that information is straightforward and easy to digest. Individuals must also be supported to understand their personal risk of COVID-19 disease, as well as the risks to the broader community. Emerging misinformation about COVID-19 vaccines receiving considerable attention should be addressed.

Mixed-methods and longitudinal approaches are needed to gather more nuanced evidence on COVID-19 vaccine acceptance as the pandemic evolves and vaccination programs expand to include COVID-19 booster vaccines, a range of vaccine choices, the next generation of COVID-19 vaccines, and vaccination of groups previously ineligible to receive COVID-19 vaccines, for example children.

Findings and recommendations presented here can inform the public health and communication responses aimed at supporting acceptance of COVID-19 vaccines, with a potential beneficial flow-on effect for other routine and seasonal immunisation programs, and future novel pandemic vaccines.
