# Supplementary material for: Factors Influencing COVID-19 Vaccine Acceptance in High Income Countries Prior to Vaccine Approval and Rollout: A Narrative Review
Source: Int J Public Health. 2022 Feb 16;67:1604221. doi: 10.3389/ijph.2022.1604221 (PMC8888412; doi:10.3389/ijph.2022.1604221)
Supplement: Supplementary file 1 [file DataSheet1.pdf]

Steffens MS, Bullivant B, Bolsewicz K, King C and Beard F (2022) Factors Influencing COVID-19 Vaccine Acceptance in High Income Countries Prior to Vaccine Approval and Rollout: A Narrative Review. Int J Public Health 67:1604221. doi: 10.3389/ijph.2022.1604221

Supplementary Material: Ovid MEDLINE search strategies

Ovid MEDLINE: COVID-19 vaccine attitudes in adults in Australia and comparable countries

Database: MEDLINE(R) All including Epub Ahead of Print, In-Process & Other Non-Indexed Citations, Daily and Versions(R) <1946-current>
Search Strategy:
----
1 exp Coronavirus/
2 exp Coronavirus Infections/
3 coronavirus$.tw.
4 ('2019 nCoV$' or 2019-nCoV$ or 2019nCoV$ or 'n CoV$' or n-CoV$ or nCoV$).tw.
5 ('covid 19' or covid-19 or covid19).tw.
6 ('SARS CoV2' or SARS-CoV2 or SARSCoV2 or SARS-CoV-2).tw.
7 1 or 2 or 3 or 4 or 5 or 6
8 exp Health Knowledge, Attitudes, Practice/
9 (attitude$ or knowledge$ or belie$ or view$ or opinion$ or thought$ or think$ or perceive$ or percepti$ or perspective$ or understand$).tw.
10 (accept$ or uptake$ or confiden$ or hesitan$).tw.
11 (barrier$ or misperception$ or information$ or misinform$ or disinform$).tw.
12 8 or 9 or 10 or 11
13 exp Vaccines/
14 (vaccin$ or immuni$).tw.
15 13 or 14
16 7 and 12 and 15
17 limit 16 to ("young adult (19 to 24 years)" or "adult (19 to 44 years)" or "young adult and adult (19-24 and 19-44)" or "middle age (45 to 64 years)" or "middle aged (45 plus years)" or "all aged (65 and over)" or "aged (80 and over)")
18 adult$.tw.
19 16 and 18
20 17 or 19
21 exp Great Britain/
22 ((united adj kingdom) or uk or britain).tw.
23 exp United States/
24 (america$ or united states).tw.
25 exp Canada/
26 canad$.tw.

27 exp New Zealand/
28 (new adj zealand$).tw.
29 exp Europe/
30 europe$.tw.
31 exp Australia/
32 australia$.tw.
33 21 or 22 or 23 or 24 or 25 or 26 or 27 or 28 or 29 or 30 or 31 or 32
34 20 and 33
35 limit 34 to yr="2020"

Ovid MEDLINE: COVID-19 vaccine attitudes in health and aged care workers

Database: MEDLINE(R) All including Epub Ahead of Print, In-Process & Other Non-Indexed Citations, Daily and Versions(R) <1946-current>
Search Strategy:
----
1 exp Coronavirus/
2 exp Coronavirus Infections/
3 coronavirus$.tw.
4 ('2019 nCoV$' or 2019-nCoV$ or 2019nCoV$ or 'n CoV$' or n-CoV$ or nCoV$).tw.
5 ('covid 19' or covid-19 or covid19).tw.
6 ('SARS CoV2' or SARS-CoV2 or SARSCoV2 or SARS-CoV-2).tw.
7 1 or 2 or 3 or 4 or 5 or 6
8 exp Health Personnel/
9 ((health$ or hospital$ or clinical$ or medical$) adj4 (worker$ or employee$ or staff or personnel)).tw.
10 ("health care worker$" or hcw or doctor$ or nurs$ or paramedic$).tw.
11 (("nursing home$" or "aged care") adj4 (worker$ or employee$ or staff or personnel)).tw.
12 8 or 9 or 10 or 11
13 7 and 12
14 exp Health Knowledge, Attitudes, Practice/
15 (attitude$ or knowledge$ or belie$ or view$ or opinion$ or thought$ or think$ or perceive$ or percepti$ or perspective$ or understand$).tw.
16 (accept$ or uptake$ or confiden$ or hesitan$).tw.
17 (barrier$ or misperception$ or information$ or misinform$ or disinform$).tw.
18 14 or 15 or 16 or 17
19 13 and 18
20 exp Vaccines/

21 (vaccin$ or immuni$).tw.
22 20 or 21
23 19 and 22
24 limit 23 to yr="2020"

Ovid MEDLINE: COVID-19 misinformation in Australia and comparable countries

Database: MEDLINE(R) All including Epub Ahead of Print, In-Process & Other Non-Indexed Citations, Daily and Versions(R) <1946-current>
Search Strategy:
----
1 exp Coronavirus/
2 exp Coronavirus Infections/
3 coronavirus$.tw.
4 ('2019 nCoV$' or 2019-nCoV$ or 2019nCoV$ or 'n CoV$' or n-CoV$ or nCoV$).tw.
5 ('covid 19' or covid-19 or covid19).tw.
6 ('SARS CoV2' or SARS-CoV2 or SARSCoV2 or SARS-CoV-2).tw.
7 1 or 2 or 3 or 4 or 5 or 6
8 exp Deception/
9 exp Mythology/
10 myth$.tw.
11 conspir$.tw.
12 (fake adj1 news).tw.
13 (falsehood$ or lie$).tw.
14 (misinform$ or disinform$).tw.
15 misperce$.tw.
16 (decept$ or decei$).tw.
17 misle$.tw.
18 8 or 9 or 10 or 11 or 12 or 13 or 14 or 15 or 16 or 17
19 7 and 18
20 exp Great Britain/
21 ((united adj kingdom) or uk or britain).tw.
22 exp United States/
23 (america$ or united states).tw.
24 exp Canada/
25 canad$.tw.
26 exp New Zealand/

27 (new adj zealand$).tw.
28 exp Europe/
29 europe$.tw.
30 exp Australia/
31 australia$.tw.
32 20 or 21 or 22 or 23 or 24 or 25 or 26 or 27 or 28 or 29 or 30 or 31
33 19 and 32
34 limit 33 to yr="2020"
